# The Effects of *P5CR* Gene Function of Endophytic Fungus *Alternaria oxytropis* OW7.8 on Swainsonine Biosynthesis

**DOI:** 10.3390/biom15040460

**Published:** 2025-03-21

**Authors:** Fan Yang, Yinzhe Li, Ping Lu, Yu Wang, Feng Gao, Bo Yuan, Ling Du, Yuling Li, Kai Jiang

**Affiliations:** 1 College of Life Science and Technology, Inner Mongolia Normal University, Hohhot 010022, China; atlas_yf@163.com (F.Y.); liyinzhe1@outlook.com (Y.L.); wangyu051606@163.com (Y.W.); imgaofeng@163.com (F.G.); yuanbo934@126.com (B.Y.); nmduling@163.com (L.D.); liyuling@163.com (Y.L.); jiangkai@imnu.edu.cn (K.J.); 2 Key Laboratory of Biodiversity Conservation and Sustainable Utilization in Mongolian Plateau for College and University of Inner Mongolia Autonomous Region, Hohhot 010022, China

**Keywords:** *Alternaria oxytropis* OW 7.8, swainsonine, *P5CR* gene

## Abstract

Locoweeds, including *Oxytropis* and *Astragalus* species, are globally recognized as plants containing swainsonine (SW), a neurotoxic alkaloid that induces neurological dysfunction and growth inhibition in livestock. SW is produced by endophytic fungi in plants; the pyrroline-5-carboxylate reductase (P5CR) gene is critical in the fungal SW biosynthetic pathway. In this study, a *P5CR* gene knockout mutant (Δ*P5CR*) was constructed from the endophytic fungus *Alternaria oxytropis* OW7.8 isolated from *Oxytropis glabra*. Compared to the wild-type strain (*A. oxytropis* OW7.8), the SW content in the Δ*P5CR* mycelia was significantly reduced, indicating that the P5CR gene plays a crucial role in promoting SW biosynthesis. Compared to the wild-type strain *A. oxytropis* OW7.8, the Δ*P5CR* mutant exhibited distinct morphological alterations in both colony and mycelial structures. The transcriptomic analysis of *A. oxytropis* OW7.8 and Δ*P5CR* revealed the downregulation of six genes associated with SW biosynthesis. Metabolomic profiling further demonstrated altered levels of six metabolites linked to SW synthesis. These findings provide foundational insights into the molecular mechanisms and metabolic pathways underlying SW biosynthesis in fungi. They hold significant value for future strategies to control SW in *Oxytropis glabra* and contribute positively to the protection and sustainable development of grassland ecosystems.

## 1. Introduction

Locoweeds, globally referred to as *Oxytropis* and *Astragalus* species containing swainsonine (SW), are predominantly distributed in western China, North America, and Australia [1,2]. SW, an indolizidine alkaloid, inhibits intracellular mannosidase activity, leading to neurological dysfunction and growth inhibition in livestock upon ingestion [3,4,5]. Notably, SW also exhibits potential as an anticancer agent by activating mitochondrial-mediated apoptosis, suppressing tumor metastasis, and inducing cancer cell death [6,7,8]. To date, studies have confirmed that SW in locoweeds is biosynthesized by endophytic fungi of the genus *Alternaria* [9,10,11,12,13].

Endophytic fungi associated with locoweeds belong to *Alternaria* spp., including four species: *A. oxytropis*, *A. cinereum*, *A. fulva*, and *A. bornmuellerii* [14,15,16]. Early work by Braun et al. isolated *Alternaria* endophytes from eight locoweed species, and Chinese researchers identified *A. oxytropis* in *Oxytropis kansuensis*, *O. glabra*, and *O. ochrocephala* [17,18,19,20,21,22]. Our research group first isolated *A. oxytropis* from *O. glabra* populations. This strain, designated *A. oxytropis* OW7.8, produces SW in vitro [23,24]. The saccharopine reductase (sac) catalyzes the following reaction in fungi: α-Aminoadipic semialdehyde + glutamate + NADPH + H^+^ ↔ saccharopine + α- ketoglutarate + NADP^+^ + H_2_O. Saccharopine is one of the intermediate compounds in SW biosynthesis. To investigate SW biosynthesis, we generated a *saccharopine reductase* gene (*sac*) knockout mutant M1 in *A. oxytropis* OW7.8 [23,24]. The M1 mutant exhibited a reduced SW level, while the complemented strain C1 had a higher SW level than both M1 and OW7.8, indicating that Sac enzyme activity promotes SW biosynthesis [23,24,25]. Furthermore, the supplementation of the culture medium with saccharopine, α-aminoadipic acid, L-lysine, or L-pipecolic acid (L-PA) enhanced SW production in both wild-type OW7.8 and M1 strains. The transcriptomic analysis of *A. oxytropis* OW7.8 and the M1 mutant revealed key genes associated with SW biosynthesis [25]. Based on these findings, it is speculated that L-PA can originate from two branches: the delta-1-piperideine-2-carboxylate (P2C) branch and delta-1-piperideine-6-carboxylate (P6C) branch [23,24,25].

A comparative genomic analysis of 34 fungal species, including locoweed-associated *Alternaria* spp., human dermatophyte *Arthroderma* spp., and entomopathogenic *Metarhizium* spp., was conducted using Hidden Markov Models (HMMs) [12,26]. This analysis identified putative SW synthesis-related gene clusters (designated *SWN* gene clusters) and predicted the functional members involved in SW biosynthesis. The *SWN* cluster comprises seven genes (*swnA*, *swnH1*, *swnH2*, *swnK*, *swnN*, *swnR*, and *swnT*; Table 1), whose encoded enzymes primarily catalyze the conversion of L-PA to SW [12,26]. Intriguingly, the composition of the SWN cluster varies across fungal species. In locoweed endophytes, the cluster contains five core genes (*swnH1*, *swnH2*, *swnK*, *swnN*, and *swnR*), whereas *swnA* and *swnT* are absent [12,26].

Our research team previously cloned the *swnN* gene (GenBank: OR596336) and the *swnH1* gene (GenBank: ON416998) from *A. oxytropis* OW7.8 [27,28]. The *swnN* and *swnH1* genes were individually knocked out in *A. oxytropis* OW7.8, and SW was undetectable in both ∆*swnN* and ∆*swnH1* mutants [27,28]. However, the complemented strain ∆*swnN/swnN* regained the ability to synthesize SW, indicating that *swnN* and *swnH1* genes promote SW biosynthesis [27,28]. Transcriptomic and metabolomic analyses of OW7.8 and ∆*swnN* predicted six genes (*sac*, *P5CR*, *swnH1*, *swnK*, *swnH2*, and *swnR*) and five metabolites (L-glutamate, α-ketoglutaric acid, L-proline, 2-aminoadipic acid, and L-PA) associated with SW biosynthesis [27]. Similarly, transcriptomic and metabolomic analyses of OW7.8 and ∆*swnH1* predicted five genes (*sac*, *swnN*, *swnK*, *swnH2*, and *swnR*) and five metabolites (α-aminoadipic acid, L-stachydrine, L-proline, saccharopine, and L-PA) linked to SW biosynthesis [28].

**Figure 1:**
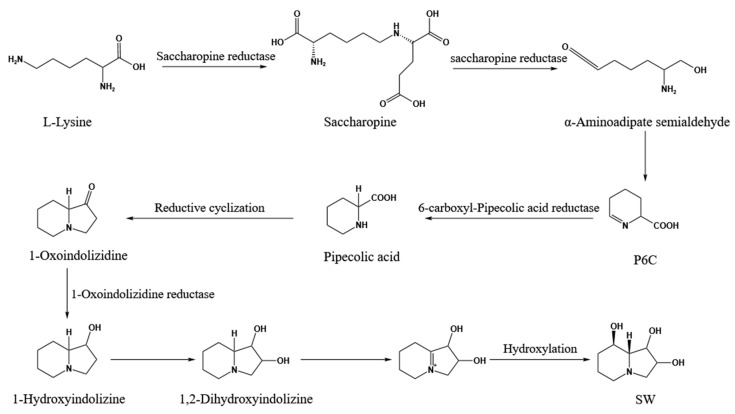
Partial biosynthesis pathways of SW from *R. leguminicola* [13].

Early investigations into fungal SW biosynthesis primarily focused on *Rhizoctonia leguminicola* [13]. In this species, the SW biosynthetic pathway proceeds as follows: L-lysine is first reduced to saccharopine, which is subsequently converted to α-aminoadipic acid semialdehyde via the action of Sac. Following conformational rearrangement, α-aminoadipic acid semialdehyde generates P6C. P6C is then reduced to L-PA by 6-carboxyl-pipecolic acid reductase. L-PA undergoes reductive cyclization to form 1-oxoindolizidine, which is further reduced by 1-oxoindolizidine reductase to yield 1-hydroxyindolizidine. Subsequent reactions produce 1,2-dihydroxyindolizidine, which is ultimately hydroxylated to form SW (Figure 1) [13].

**Figure 2:**
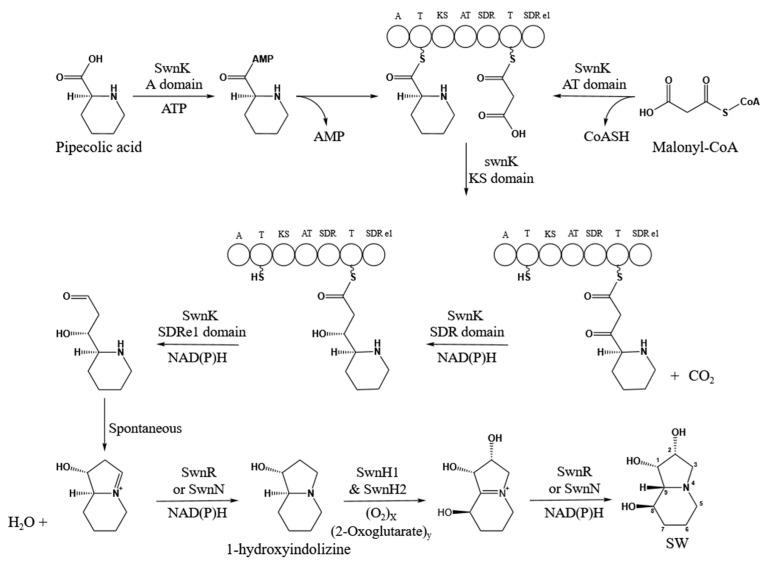
Partial biosynthesis pathways of SW in *M. robertsii* [12].

In *Metarhizium robertsii*, the proposed biosynthetic pathway from L-PA to SW involves sequential enzymatic steps mediated by the *SWN* gene cluster [12]. L-PA and malonyl-CoA are initially condensed by the multifunctional protein SwnK, followed by reductive modifications catalyzed by SwnR or SwnN, yielding 1-hydroxyindolizidine intermediates. Subsequent hydroxylation by SwnH1 and SwnH2, coupled with further reduction by SwnR/SwnN, ultimately generates SW (Figure 2) [12].

**Figure 3:**
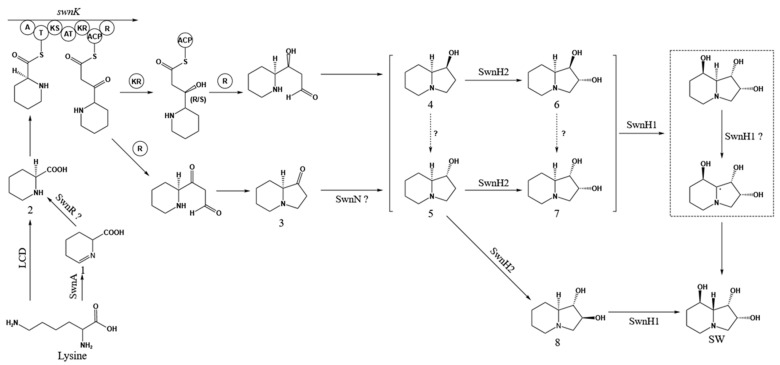
Biosynthesis pathways of SW in *M. robertsii* [29].

To delineate the roles of *SWN* cluster members, knockout experiments were performed in *M. robertsii* [29]. These studies revealed two potential pathways for L-PA biosynthesis from L-lysine: L-Lysine is converted to P6C via the lysine aminotransferase (LAT/SwnA), which is subsequently reduced to L-PA by the reductase SwnR. L-Lysine undergoes direct cyclization to L-PA through lysine cyclodeaminase (LCD). The L-PA-derived intermediates then converge into a unified pathway: SwnK facilitates the condensation of L-PA with malonyl-CoA, generating (8aS)-1-oxoindolizidine. This intermediate is reduced by SwnN to form stereoisomers (1S,8aS)-1-hydroxyindolizidine and (1R,8aS)-1-hydroxyindolizidine. SwnH2 catalyzes the hydroxylation of these isomers, producing (1R,2S,8aS)-1,2-dihydroxyindolizidine, (1S,2S,8aS)-1,2-dihydroxyindolizidine, or (1S,2R,8aS)-1,2-dihydroxyindolizidine. Finally, 1,2-dihydroxyindolizine undergoes hydroxylation to form SW (Figure 3) [29].

The *P5CR* gene (pyrroline-5-carboxylate reductase gene, *P5CR*) encodes pyrroline-5-carboxylate reductase (P5CR), which catalyzes the conversion of P6C to L-PA in fungi [30,31]. L-PA is recognized as a critical precursor for SW biosynthesis and serves as a key intermediate in the secondary metabolism of plants and microorganisms [32,33]. Notably, a limited number of studies have proposed that the *P5CR* gene in *A. oxytropis* from *O. ochrocephala* may also belong to the SWN cluster [21]. The transcriptomic analysis of OW7.8 and the M1 mutant revealed the downregulation of the *P5CR* gene in M1 compared to the wild-type strain, suggesting a potential regulatory role of *P5CR* in SW biosynthesis [23]. Our team previously cloned the *P5CR* gene from the endophytic fungus *Alternaria oxytropis* OW7.8 (GenBank accession number: ON004912).

This study aims to investigate the role of the *P5CR* gene in SW biosynthesis in *Alternaria oxytropis* OW7.8 by constructing a *P5CR* knockout mutant (Δ*P5CR*). The SW levels in both wild-type *A. oxytropis* OW7.8 and the Δ*P5CR* mutant will be quantified, followed by comprehensive transcriptomic and metabolomic analyses. These experiments are designed to elucidate the functional contribution of *P5CR* to the SW biosynthetic pathway in *A. oxytropis* OW7.8 and to refine the overall understanding of SW biosynthesis in this endophytic fungus. The findings of this research will provide critical insights into the molecular mechanisms and metabolic pathways underlying the fungal SW synthesis. Furthermore, this study holds significant practical value for guiding SW control strategies in locoweeds and contributes positively to the conservation and sustainable management of grassland ecosystems.

## 2. Materials and Methods

### 2.1. Fungal Strain

The *A. oxytropis* OW7.8 strain was isolated from *O. glabra* by our research group [34]. The mycelia were cultured on potato dextrose agar (PDA) medium at 25 °C for subsequent experiments.

### 2.2. Genomic DNA Extraction from A. oxytropis OW7.8

Genomic DNA was extracted from *A. oxytropis* OW7.8 using a plant genomic DNA extraction kit (TIANGEN). The quality of the extracted DNA was assessed by 1% agarose gel electrophoresis and quantified using a Q5000 spectrophotometer (Quawell).

### 2.3. Construction of the P5CR Gene Knockout Vector

The upstream and downstream homologous sequences of the *P5CR* gene were amplified using *A. oxytropis* OW7.8 genomic DNA as the template and P5CR-UF/P5CR-UR (5′-CGCGGATCCATGGCAAACACGCAGGAATCTAAGC-3′; 5′-GCACCGGTGTCAC AGGCGATGAAGTTGGAGAG-3′) and P5CR-DF/P5CR-DR (5′-TATCCTGCAGGCTC AGCGAGGCGCAGTACAAGCTT-3′; 5′-CGGAATTCCTACCGCCTGTGGTTCACGA-3′) as primers. The hygromycin phosphotransferase gene (*hpt*) was amplified using the pCT74 plasmid (MiaoLingBio) as the template and H-F/H-R (5′-GCACCGGTGGCTTGGCTGGAGCTAGTGGAGGTC-3′; 5′-CGGCCTGCAGGGAACCCGCGGTCGGCATCTACTCTAT-3′) as primers. Using restriction enzyme digestion and ligation, the upstream and downstream homologous sequences of *P5CR* were flanked to the *hpt* gene, constructing the *P5CR* knockout cassette. This cassette was then ligated into the pUC19 vector (Takara) to generate the *P5CR* gene knockout vector.

### 2.4. Protoplast Preparation and Transformation of A. oxytropis OW7.8

Young mycelia of *A. oxytropis* OW7.8 were inoculated into 150 mL of potato dextrose broth (PDB) medium and cultured at 180 rpm and 25 °C for approximately 5 days. The protoplast lysis solution was prepared by dissolving 0.3 g Driselase, 0.1 g Lysing Enzyme, and 0.004 g Chitinase in a 15 mL centrifuge tube containing a small volume of 1.2 mol/L MgSO_4_ solution, which was then adjusted to a final volume of 10 mL. The mixture was shaken at 80 rpm and 30 °C for 30 min and filtered through a 0.22 μm organic filter into a 50 mL centrifuge tube. Mycelial balls were filtered through a Miracloth (22–25 μm pore size) funnel, rinsed twice with ddH_2_O and once with 1.2 mol/L MgSO_4_, and gently squeezed before being added to the lysis solution. The mixture was incubated at 80 rpm and 30 °C for 4 h. After incubation, the solution was filtered through Miracloth into a new 50 mL centrifuge tube, rinsed twice with 1.2 mol/L MgSO_4_, and centrifuged at 4000 rpm for 5 min. The supernatant was discarded, and the pellet was resuspended in 5 mL of STC buffer (1 mol/L Sorbitol, 100 mmol/L CaCl_2_, 100 mmol/L Tris-HCl), followed by centrifugation at 4000 rpm for 5 min. The pellet was resuspended in an appropriate volume of STC buffer, and the protoplasts were aliquoted into 100 μL portions. Approximately 2 μg of the *P5CR* knockout vector was added to each tube, mixed gently, and incubated on ice for 25 min. SPTC buffer (prepared fresh by adding 0.4 g PEG8000 to 1 mL STC) was filter-sterilized using a 0.22 μm organic filter. One milliliter of SPTC buffer was added to each tube, mixed gently, and incubated at room temperature for 30 min. After centrifugation at 4000 rpm for 5 min, the supernatant was discarded. Each tube was mixed with 12.5 mL of molten 1.5% TB3 regeneration medium(1mol/L Sucrose, 0.3% Yeast Extract, 0.3% Casein, 1.5% Agarose L.M.P) containing 50 μg/mL ampicillin (Amp) and 1 μg/mL hygromycin B (Hyg B), poured into plates, and incubated overnight at 25°C. Molten 0.7% TB3 regeneration medium (1mol/L Sucrose, 0.3% Yeast Extract, 0.3% Casein, 0.7% Agarose L.M.P) supplemented with 50 μg/mL Amp and 2 μg/mL Hyg B was added to the plates and incubated at 25 °C for approximately 14 days.

### 2.5. Screening and Identification of Transformants

Transformants resistant to Hyg B were screened and verified by PCR. Genomic DNA from transformants was used as the template for amplification with the following primer pairs: hptF/hptR (5′-GGCTTGGCTGGAGCTAGTGGAGGTC-3′; 5′-GAACCCGCGGTCGGCATCTACTCTAT-3′) for the *hpt* sequence, P5CR-TF/hptR (5′-ATCCGAACCGTCCAAG-3′; 5′-GAACCCGCGGTCGGCATCTACTCTAT-3′) for the upstream homologous sequence plus *hpt*, P5CR-TF/P5CR-TR (5′-ATCCGAACCGTCCAAG-3′; 5′-AAGTCCAGTGGTTCCTCTCAT-3′) for the *P5CR* knockout cassette, and HYP-F/HYP-R (5′-GACATCGTGCTGCTGAGTT-3′; 5′-GGTCCATCGGGATACAAG-3′) for the internal *P5CR* sequence. PCR products were analyzed by gel electrophoresis and sequenced for validation (Sangon Biotech, Shanghai, China).

### 2.6. Scanning Electron Microscopy of Mycelia

The colony morphology of OW7.8 and Δ*P5CR* after 20 days of culture was observed. Mycelial morphology was examined using a scanning electron microscope (SU8100, 3.0 kV, ×30).

### 2.7. Extraction and Detection of SW in A. oxytropis OW7.8 and ΔP5CR Mycelia

Mycelia from 20-day cultures of *A. oxytropis* OW7.8 and Δ*P5CR* were extracted using an acetic acid–chloroform solution. SW was purified using cation exchange resin and eluted with 1 mol/L ammonia. SW levels were quantified by HPLC-MS, with three replicates per sample. Data were analyzed using one-way ANOVA in GraphPad Prism 9.5.

### 2.8. Transcriptome Sequencing and Analysis of A. oxytropis OW7.8 and ΔP5CR

Mycelia from 20-day cultures of *A. oxytropis* OW7.8 and Δ*P5CR* were flash-frozen in liquid nitrogen and stored on dry ice for sequencing. Three biological replicates were prepared for each strain. Transcriptome sequencing was performed on the Illumina NovaSeq 6000 platform (Novogene, Beijing, China). Data analysis was conducted using the R packages DESeq2 and clusterProfiler, with differentially expressed genes (DEGs, |log_2_(Fold Change)| ≥ 1 and padj ≤ 0.05) subjected to KEGG and GO functional enrichment analysis [35,36,37].

### 2.9. Metabolomic Profiling and Analysis of A. oxytropis OW7.8 and ΔP5CR

Mycelia from 20-day cultures of *A. oxytropis* OW7.8 and Δ*P5CR* were flash-frozen in liquid nitrogen and stored on dry ice for metabolomic analysis. Five biological replicates were prepared for each strain. Metabolites were detected using HPLC-MS^2^ technology (Novogene, Beijing, China). Data analysis was performed using the R package Compound Discoverer, and differentially metabolites (VIP > 1, |log_2_(Fold Change)| ≥ 0.59 and *p*-value < 0.05) were subjected to KEGG functional enrichment analysis [38,39,40].

### 2.10. Extraction of SW Biosynthesis-Related Gene Expression Levels in A. oxytropis OW7.8 and ΔP5CR

Total RNA was extracted from *A. oxytropis* OW7.8 and Δ*P5CR* using the OminiPlant RNA Kit (CWBIO) and reverse-transcribed into cDNA. RT-qPCR was performed using cDNA templates from both strains, with the actin gene as the internal reference. The following primer pairs were used to amplify six SW biosynthesis-related genes: sac-F/sac-R (5′-CTGCTGCTCGGTGCTGGATTC-3′; 5′-CTAGACTGATGGCGTTGGTGTTGG-3′); R-F/R-R (5′-TTCTACTTTGCCACACACGAACCC-3′; 5′-ATAGTCAGCCAACCAGCCAATGC-3′); K-F/K-R (5′-GACCGCTTGCTCGCCTGTG-3′; 5′-CTCGTCAACTCGTCCAACACTTCC-3′); N-F/N-R (5′-TGACTAAGTTCATTCCCAGCG-3′; 5′-AAGAGTTCCGTTTCTGCCTC-3′); H2-F/H2-R (5′-CATCTGCTCCTCGCTTGCTACC-3′; 5′-CAGGACAACGCCTCCATCTCTTTC-3′); H1-F/H1-R (5′-TTGCTTTGCGGAGATGGAACCAG-3′; 5′-CGGAGTGTGCCTGAGATGAAGAAG-3′). Gene expression levels were statistically analyzed using GraphPad Prism 9.5.0, with one-way ANOVA applied to compare data across sample groups.

## 3. Results

### 3.1. Construction of the P5CR Gene Knockout Vector in A. oxytropis

**Figure 4:**
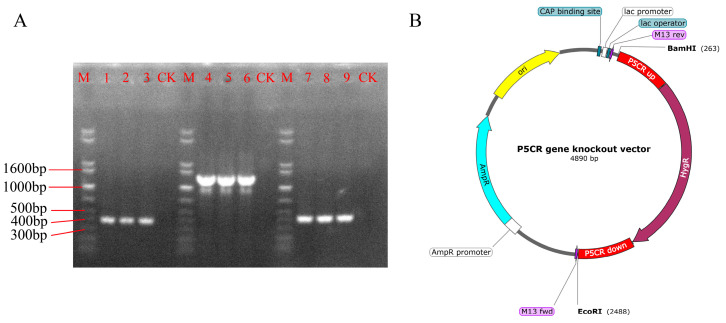
Construction of the *P5CR* gene knockout vector. Note: M: 1 kb plus DNA ladder. (**A**) PCR electrophoresis image of the upstream and downstream homologous sequences of the *P5CR* gene and the *hpt* gene. Lane 1, 2, 3: Upstream homologous sequence of the *P5CR* gene, expected product: 422 bp. Lane 4, 5, 6: *hpt* gene sequence, expected product: 1398 bp. Lane 7, 8, 9: Downstream homologous sequence of the *P5CR* gene, expected product: 439 bp. CK: Negative control. (**B**) Schematic diagram of the *P5CR* gene knockout vector. Original Images for Gels see Supplementary Materials.

The upstream and downstream homologous sequences of the *P5CR* gene and the *hpt* gene were successfully amplified (Figure 4A). These three fragments were ligated to construct the *P5CR* knockout cassette, which was then inserted into the pUC19 vector to generate the *P5CR* knockout vector (Figure 4B). The final vector contained the ampicillin resistance gene (*Amp^R^*) and the *P5CR* knockout cassette, which included the *hpt* gene.

### 3.2. Screening and Identification of Knockout Transformants

**Figure 5:**
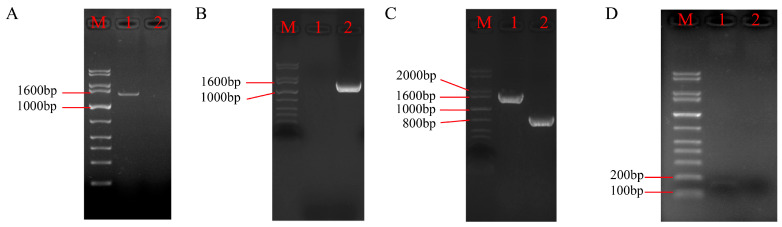
PCR electrophoresis images of transformants. Note: M: 1 kb plus DNA ladder (**A**) PCR amplification of the *hpt* gene sequence, expected product: 1380 bp. Lane 1: Transformant DNA; Lane 2: Wild-type strain DNA. (**B**) PCR amplification of the upstream homologous sequence of the *P5CR* gene + *hpt* gene sequence, expected product: 1563 bp. Lane 1: Wild-type strain DNA; Lane 2: Transformant DNA. (**C**) PCR amplification of the *P5CR* gene knockout cassette sequence, expected product: 759 bp for the wild-type strain and 1799 bp for the knockout strain. Lane 1: Transformant DNA; Lane 2: Wild-type strain DNA. (**D**) PCR amplification of the internal sequence of the *P5CR* gene, expected product: 152 bp. Lane 1: Wild-type strain DNA; Lane 2: Transformant DNA. Original Images for Gels see Supplementary Materials.

One week after transforming the *A. oxytropis* OW7.8 protoplasts with the knockout vector, a small number of transformants appeared on the TB3 medium. These transformants were transferred to a PDA medium supplemented with 2 μg/mL of hygromycin B (Hyg B) for resistance screening. The PCR analysis confirmed that the knockout strain Δ*P5CR* could amplify the *hpt* gene, the upstream homologous sequence of *P5CR* plus the *hpt* gene, and the *P5CR* knockout cassette, but not the internal sequence of *P5CR* (Figure 5). The sequencing of the PCR products further validated the correct integration of the knockout cassette, confirming the successful generation of the Δ*P5CR* mutant.

### 3.3. Comparison of Colony Morphology and Scanning Electron Microscopy (SEM) Structures Between A. oxytropis OW7.8 and ΔP5CR

**Figure 6:**
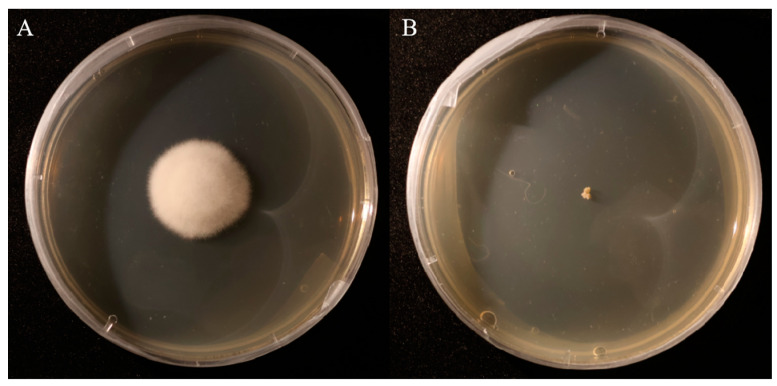
Colonies of *A. oxytropis* OW7.8 and Δ*P5CR*. Note: (**A**) *A. oxytropis* OW7.8 (**B**) Δ*P5CR.*

The wild-type *A. oxytropis* OW7.8 exhibited white, fluffy colonies with a raised center and radial mycelial growth, accompanied by melanin accumulation on the reverse side. In contrast, the Δ*P5CR* mutant formed raised, irregularly edged colonies with a creamy-white to pale-yellow appearance, lacking pigment accumulation. The mutant also displayed slower growth, irregular mycelial patterns, and densely packed, stacked hyphae (Figure 6).

**Figure 7:**
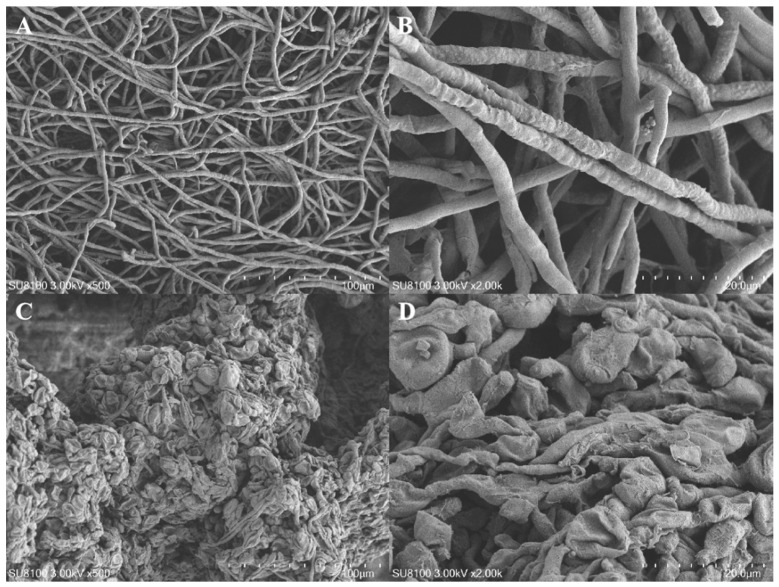
SEM Images of *A. oxytropis* OW7.8 and Δ*P5CR*. Note: (**A**) SEM image of *A. oxytropis* OW7.8 (500×). (**B**) SEM image of *A. oxytropis* OW7.8 (2000×). (**C**) SEM image of Δ*P5CR* (500×). (**D**) SEM image of Δ*P5CR* (2000×).

The SEM analysis revealed distinct morphological differences between the two strains. The wild-type *A. oxytropis* OW7.8 exhibited typical fungal mycelial structures, with loose, filamentous, and well-organized hyphae (Figure 7A,B). In contrast, the Δ*P5CR* mutant showed an abnormal mycelial morphology, characterized by tightly packed, swollen, and irregularly shaped hyphae, with some structures appearing shrunken or collapsed (Figure 7C,D).

### 3.4. SW Levels in A. oxytropis OW7.8 and ΔP5CR Mycelia

**Figure 8:**
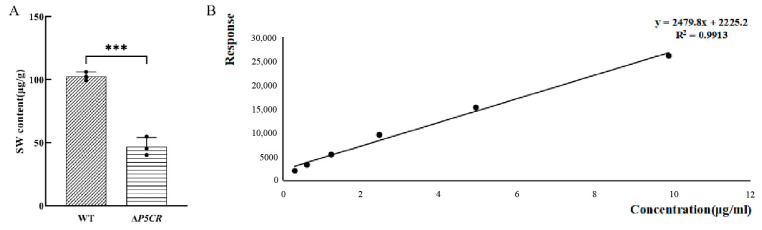
SW Levels in *A. oxytropis* OW7.8 and Δ*P5CR*. Note: (**A**) SW levels in mycelia (***: *p* < 0.001). (**B**) Standard curve for SW level detection, with the standard equation: y = 2479.8x + 2225.2, R^2^ = 0.9913.

The SW content in 20-day-old mycelia of *A. oxytropis* OW7.8 was 102.69 ± 3.38 μg/g·DW, while that in Δ*P5CR* was significantly lower at 46.82 ± 7.41 μg/g·DW (*p* < 0.001) (Figure 8).

### 3.5. Transcriptomic Analysis of A. oxytropis OW7.8 and ΔP5CR

**Figure 9:**
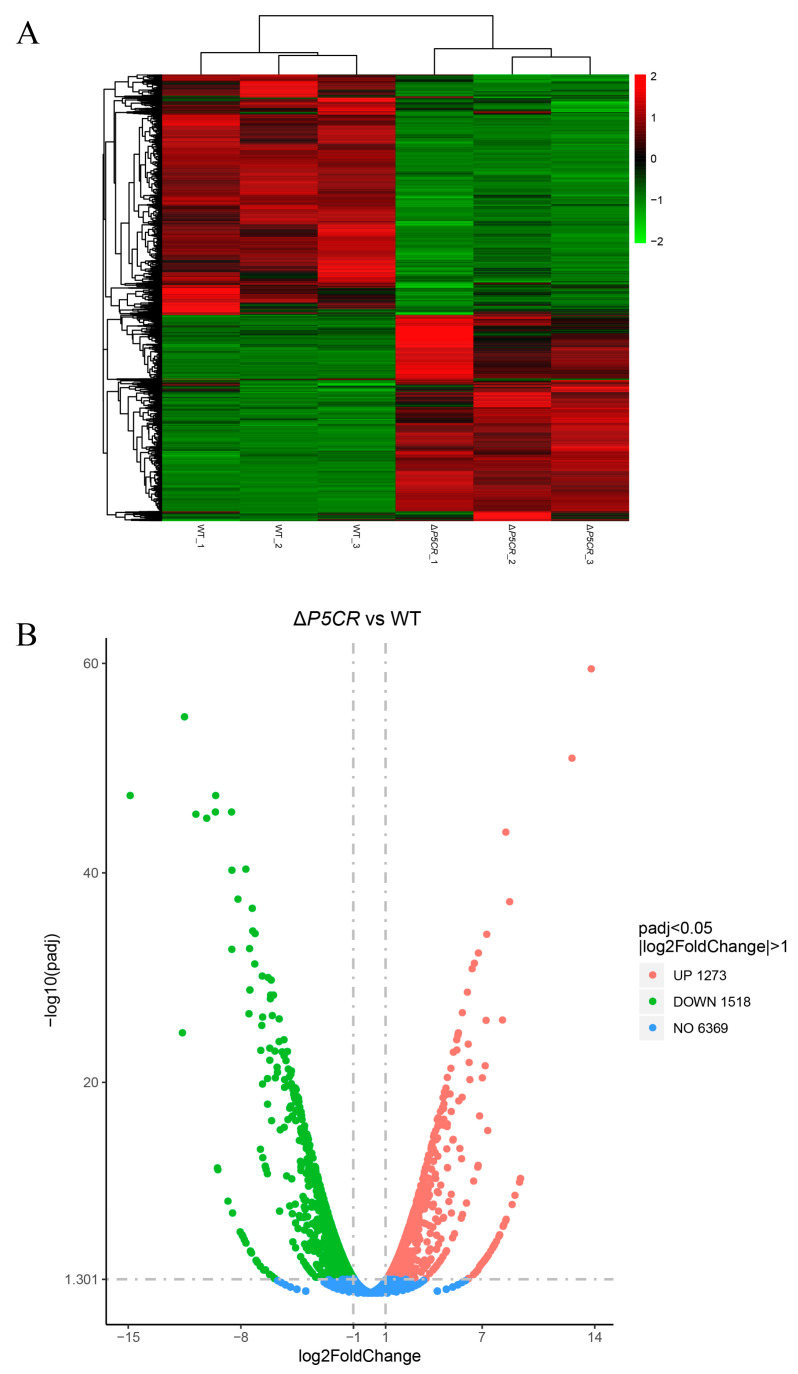
Transcriptome analysis of *A. oxytropis* OW7.8 and Δ*P5CR*. Note: (**A**) Heatmap of clustered DEGs between the two groups. (**B**) Volcano plot of differential comparisons.

Transcriptome sequencing of *A. oxytropis* OW7.8 and Δ*P5CR* yielded 252,427,347 clean reads. Significant expression differences were observed between OW7.8 and Δ*P5CR* (Figure 9A), with 2791 differentially expressed genes (DEGs) identified, including 1273 upregulated and 1518 downregulated genes (Figure 9B).

**Figure 10:**
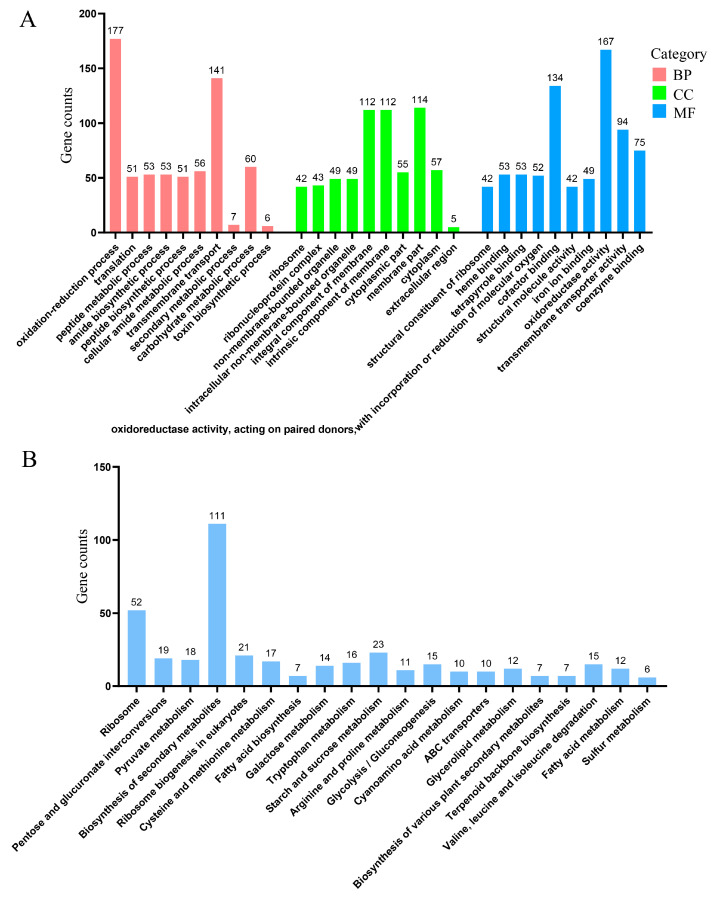
Transcriptome analysis of *A. oxytropis* OW7.8 and Δ*P5CR*. Note: (**A**) GO functional classification annotation. (**B**) KEGG enrichment analysis.

The GO enrichment analysis annotated 1535 DEGs to 582 biological processes (BPs), 183 cell components (CCs), and 770 molecular functions (MFs). Key enriched processes included an oxidation-reduction process, transmembrane transport, membrane part, intrinsic component of membrane, oxidoreductase activity, and cofactor binding (Figure 10A). Specifically, for the oxidation-reduction process, 55 genes were upregulated and 122 genes were downregulated; for oxidoreductase activity, 50 genes were upregulated and 117 genes were downregulated; for transmembrane transport, 41 genes were upregulated and 100 genes were downregulated; for cofactor binding, 47 genes were upregulated and 87 genes were downregulated; and for transmembrane transporter activity, 25 genes were upregulated and 69 genes were downregulated.

The KEGG enrichment analysis annotated 893 DEGs, with the largest proportions associated with secondary metabolism and ribosome biosynthesis. Key pathways included ribosome, starch and sucrose metabolism, ribosome biogenesis in eukaryotes, and pentose and glucuronate interconversions (Figure 10B). Specifically, for the biosynthesis of secondary metabolites, 44 genes were upregulated and 67 genes were downregulated. For the ribosome, fifty-one genes were upregulated and one gene was downregulated. For starch and sucrose metabolism: five genes upregulated and eighteen genes were downregulated. For ribosome biogenesis in eukaryotes, twenty genes were upregulated and one gene was downregulated. For pentose and glucuronate interconversions, three genes were upregulated and sixteen genes were downregulated.

Notably, the expression levels of *swnN*, *swnR*, *swnK*, *swnH1*, *Aminoadipate reductase*, and sac were downregulated in Δ*P5CR*.

### 3.6. Metabolomic Profiling of A. oxytropis OW7.8 and ΔP5CR

**Figure 11:**
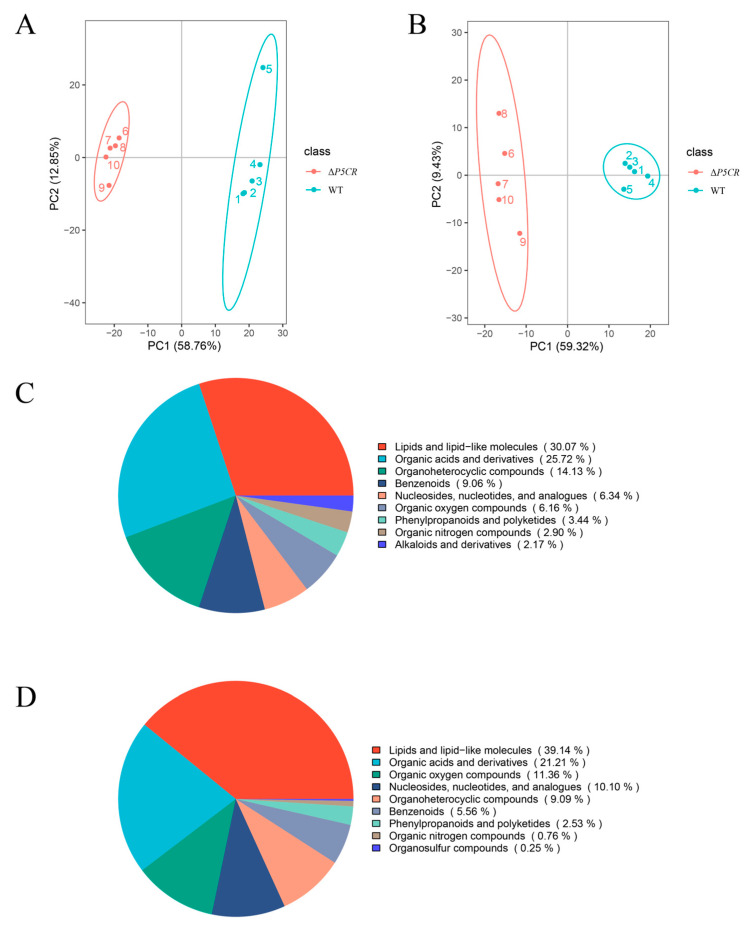
Metabolomic analysis of *A. oxytropis* OW7.8 and Δ*P5CR*. Note: (**A**) Principal component analysis (PCA) in positive ion mode. (**B**) Principal component analysis (PCA) in negative ion mode. (**C**) Metabolite classification pie chart in positive ion mode. (**D**) Metabolite classification pie chart in negative ion mode.

**Figure 12:**
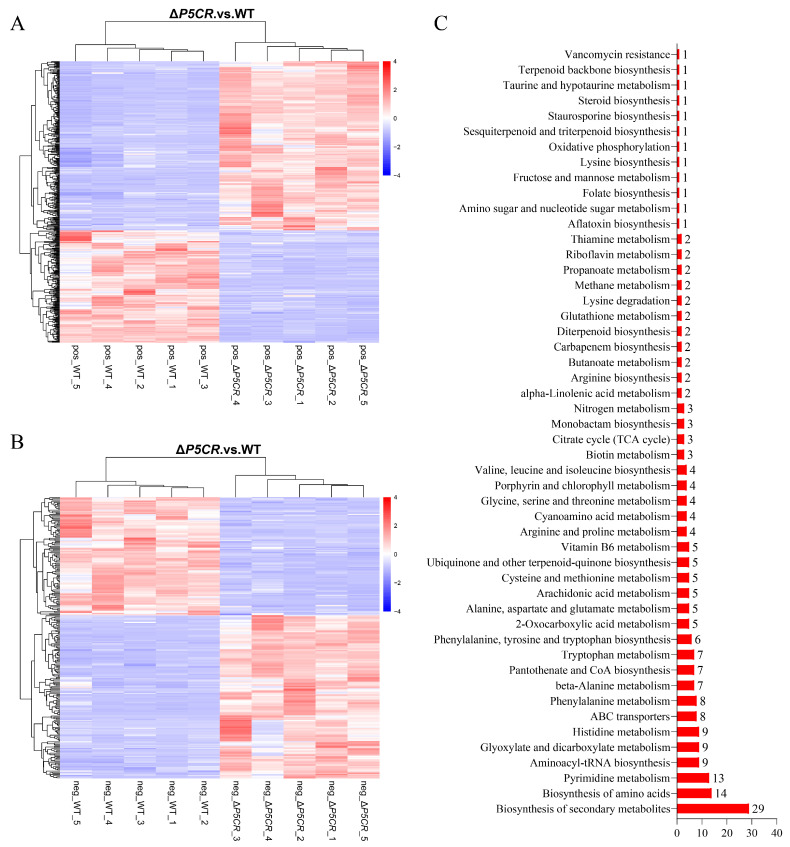
Metabolomic analysis of *A. oxytropis* OW7.8 and Δ*P5CR*. Note: (**A**) Clustering heatmap analysis of differential metabolites in positive ion mode. (**B**) Clustering heatmap analysis of differential metabolites in negative ion mode. (**C**) KEGG enrichment analysis of differential metabolites.

The principal component analysis (PCA) of metabolomic data revealed distinct metabolite profiles between Δ*P5CR* and OW7.8 (Figure 11A,B). In positive ion mode, the most abundant metabolites were lipids and lipid-like molecules (30.07%), followed by phenylpropanoids and polyketides (3.44%) and alkaloids and derivatives (2.17%). In negative ion mode, lipids and lipid-like molecules accounted for 39.14%, while phenylpropanoids and polyketides represented 2.53% (Figure 11C,D). A total of 498 differentially expressed metabolites (317 upregulated, 191 downregulated) were detected in positive ion mode, and 269 (159 upregulated, 110 downregulated) were detected in negative ion mode (Figure 12A,B).

The KEGG enrichment analysis of 777 differential metabolites annotated 222 metabolites to 50 metabolic pathways. These include the biosynthesis of secondary metabolites (twenty-nine), biosynthesis of amino acids (fourteen), Pyrimidine metabolism (thirteen), Aminoacyl-tRNA biosynthesis (nine), and Glyoxylate and dicarboxylate metabolism (nine) (Figure 12C). Six metabolites closely associated with SW biosynthesis were identified: saccharopine, L-PA, α-aminoadipic acid, L-lysine, L-proline, and L-glutamate. Among these, saccharopine, L-PA, α-aminoadipic acid, L-lysine, and L-proline were downregulated, while L-glutamate was upregulated.

### 3.7. Expression of SW Biosynthesis-Related Genes in A. oxytropis OW7.8 and ΔP5CR

**Figure 13:**
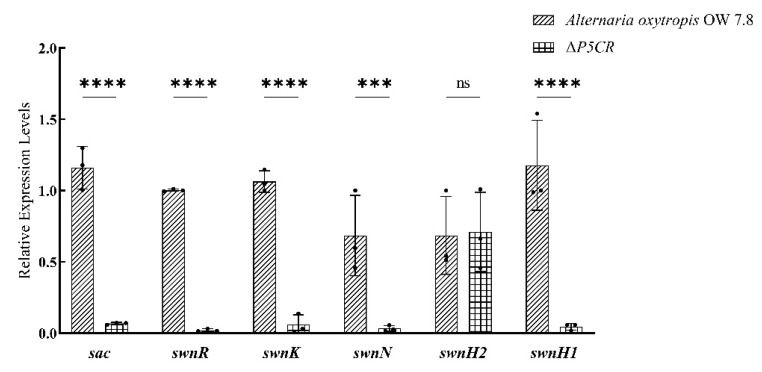
Expression of SWN gene cluster members and sac Gene in *A. oxytropis* OW7.8 and Δ*P5CR*. Note: X-axis: gene names; Y-axis: relative gene expression levels; significance levels: *** *p* < 0.0001, **** *p* < 0.00001, ns *p* >0.05.

The RT-qPCR analysis of 20-day-old cultures revealed that the expression levels of *sac*, *swnR*, *swnK*, *swnN*, and *swnH1* were significantly lower in Δ*P5CR* compared to *A. oxytropis* OW7.8 (*p* < 0.001). No significant difference was observed in the expression of *swnH2* (Figure 13).

## 4. Discussion

This study investigated the role of the *P5CR* gene in SW biosynthesis in the locoweed endophytic fungus *A. oxytropis* OW7.8. For the first time, we successfully knocked out the *P5CR* gene in *A. oxytropis* OW7.8, resulting in a significant reduction in SW levels in the Δ*P5CR* mutant compared to the wild-type strain. This finding indicates that P5CR enzyme activity promotes SW biosynthesis in fungi. Additionally, the Δ*P5CR* mutant exhibited slower growth, lack of pigment accumulation, irregular colony edges, and densely packed, swollen hyphae with partially shrunken and collapsed structures. These observations suggest that P5CR also influences the growth, reproduction, and metabolic processes of *A. oxytropis* OW7.8.

Transcriptomic data suggest that SW biosynthesis in *A. oxytropis* OW7.8 is associated with pathways related to alkaloid and derivative metabolism, organic nitrogen compound metabolism, heterocyclic compound metabolism, terpenoid and polyketide metabolism, arginine and proline metabolism, secondary metabolite biosynthesis, and lysine biosynthesis and degradation. Among the DEGs in *A. oxytropis* OW7.8 and Δ*P5CR*, six genes closely associated with SW biosynthesis (*swnN*, *swnR*, *swnK*, *swnH1*, *Aminoadipate reductase*, and *sac*) were downregulated. The products of *swnN*, *swnR*, *swnK*, and *swnH1* catalyze reactions downstream of P5CR. The knockout of *P5CR* likely reduced L-PA formation, leading to the downregulation of these four genes. However, due to limitations in transcriptomic platforms, genes such as *AASS*, *L-lysyl-alpha-oxidase*, *lysDH*, and *Saccharopine oxidase* were not enriched.

The metabolomic analysis identified six metabolites closely associated with SW biosynthesis: saccharopine, L-PA, α-aminoadipic acid, L-lysine, L-proline, and L-glutamate. Among these, saccharopine, L-PA, α-aminoadipic acid, L-lysine, and L-proline were downregulated in Δ*P5CR*. Saccharopine, α-aminoadipic acid, L-PA, and L-lysine are key precursors in the SW biosynthetic pathway. The inactivation of P5CR in Δ*P5CR* disrupted the conversion of P6C to L-PA, leading to reduced SW levels and the downregulation of upstream metabolites (saccharopine, α-aminoadipic acid, and L-lysine). P5CR also catalyzes the synthesis of L-proline from P5C. In Δ*P5CR*, the inability to convert P5C to L-proline resulted in reduced L-proline levels, while the glutamate-semialdehyde dehydrogenase-mediated conversion of P5C to L-glutamate led to upregulated L-glutamate levels. Additionally, other enzymes, such as SwnR, may participate in the conversion of P6C to L-PA. However, due to limitations in metabolite databases, intermediates such as 6-amino-2-oxohexanoate, P2C, and P6C were not detected.

Integrated transcriptomic and metabolomic analyses revealed that the downregulation of *Aminoadipate reductase* reduced α-aminoadipic acid levels, as this enzyme catalyzes the conversion of α-aminoadipic acid to α-aminoadipate semialdehyde. Similarly, the downregulation of *sac*, which catalyzes the synthesis of saccharopine from α-aminoadipate semialdehyde, may be attributed to reduced levels of this intermediate. The SW biosynthetic pathway predicted based on the transcriptomic and metabolomic data in this study is consistent with the pathway proposed in our latest research [27,28]. These findings deepen our understanding of the SW biosynthetic pathway in *A. oxytropis* OW7.8 and provide insights into the enzymatic reactions involved. In the future, as more SW biosynthetic genes are characterized, we hope to further elucidate and refine the SW biosynthetic pathway.

## 5. Conclusions

This study constructed the gene knockout mutant strain Δ*P5CR* of *A. oxytropis* OW7.8. Compared to OW7.8, the SW content in Δ*P5CR* was significantly reduced, indicating that the *P5CR* gene promotes SW biosynthesis. The mutant also exhibited an altered colony and mycelial morphology. Transcriptomic and metabolomic analyses of *A. oxytropis* OW7.8 and Δ*P5CR* identified six downregulated genes and six differentially expressed metabolites closely associated with SW biosynthesis. The predicted SW biosynthetic pathway based on these omics data is consistent with our recent findings. This research provides a foundation for elucidating the molecular mechanisms and metabolic pathways of SW biosynthesis in fungi, offering valuable insights for controlling SW in *Oxytropis glabra* and contributing to the conservation and sustainable management of grassland ecosystems.

## Figures and Tables

**Table 1:** Members of the SWN gene clusters and their predicted functions [12,26].

Gene	Encoding Product	Function Prediction
*swnA*	Aminotransferase	Catalyzing the synthesis of pyrroline-6-carboxylate (P6C) from *L*-lysine
*swnR*	Dehydrogenase or reductase	Catalyzing the synthesis of *L*-PA from P6C
*swnK*	Multifunctional protein	Catalyzing the synthesis of 1-oxoindolizidine (or 1-hydroxyindolizine) from *L*-PA
*swnN*	Dehydrogenase or reductase	Catalyzing the synthesis of 1-hydroxyindolizine from 1-oxoindolizidine
*swnH1*	Fe(II)/α-Ketoglutarate-dependent dioxygenase	Catalyzing the synthesis of SW from 1,2-dihydroxyindolizine
*swnH2*	Fe(II)/α-Ketoglutarate-dependent dioxygenase	Catalyzing the synthesis of 1,2-dihydroxyindolizine form 1-hydroxyindolizine
*swnT*	Transmembrane transporter	Transport of SW

## Data Availability

The data presented in this study are available on reasonable request from the corresponding author.

## Supplementary Materials

The following supporting information can be downloaded at: https://www.mdpi.com/article/10.3390/biom15040460/s1.
