# Supplementary material for: The Effects of P5CR Gene Function of Endophytic Fungus Alternaria oxytropis OW7.8 on Swainsonine Biosynthesis
Source: Biomolecules. 2025 Mar 21;15(4):460. doi: 10.3390/biom15040460 (PMC12025043; doi:10.3390/biom15040460)

Figure 4A

Original Images for Gels

PCR products of upstream and downstream homologous sequences of the *P5CR* gene and the *hpt* gene by electrophoresis

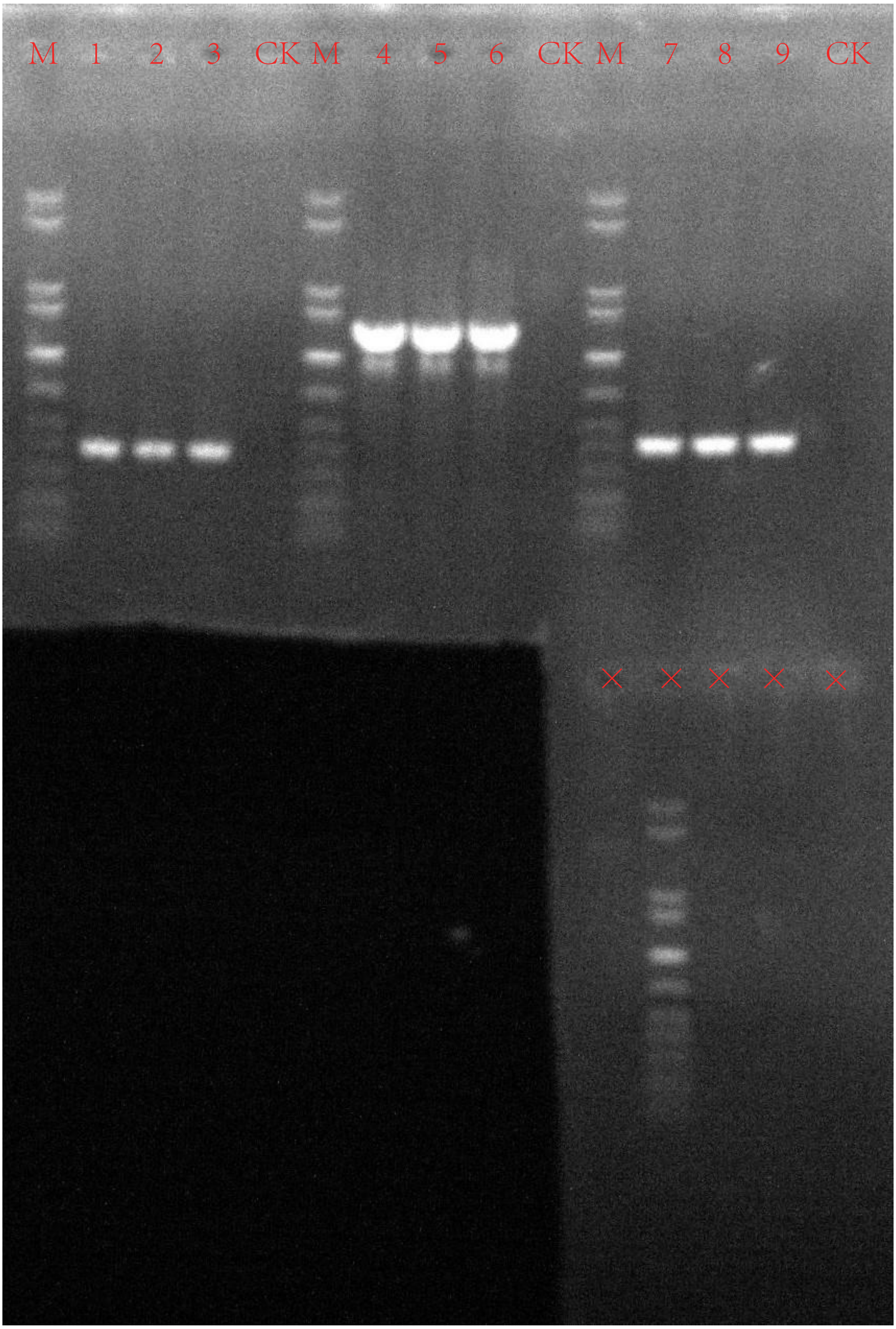

Figure 5A

Original Images for Gels

PCR products of *hpt* gene sequence by electrophoresis

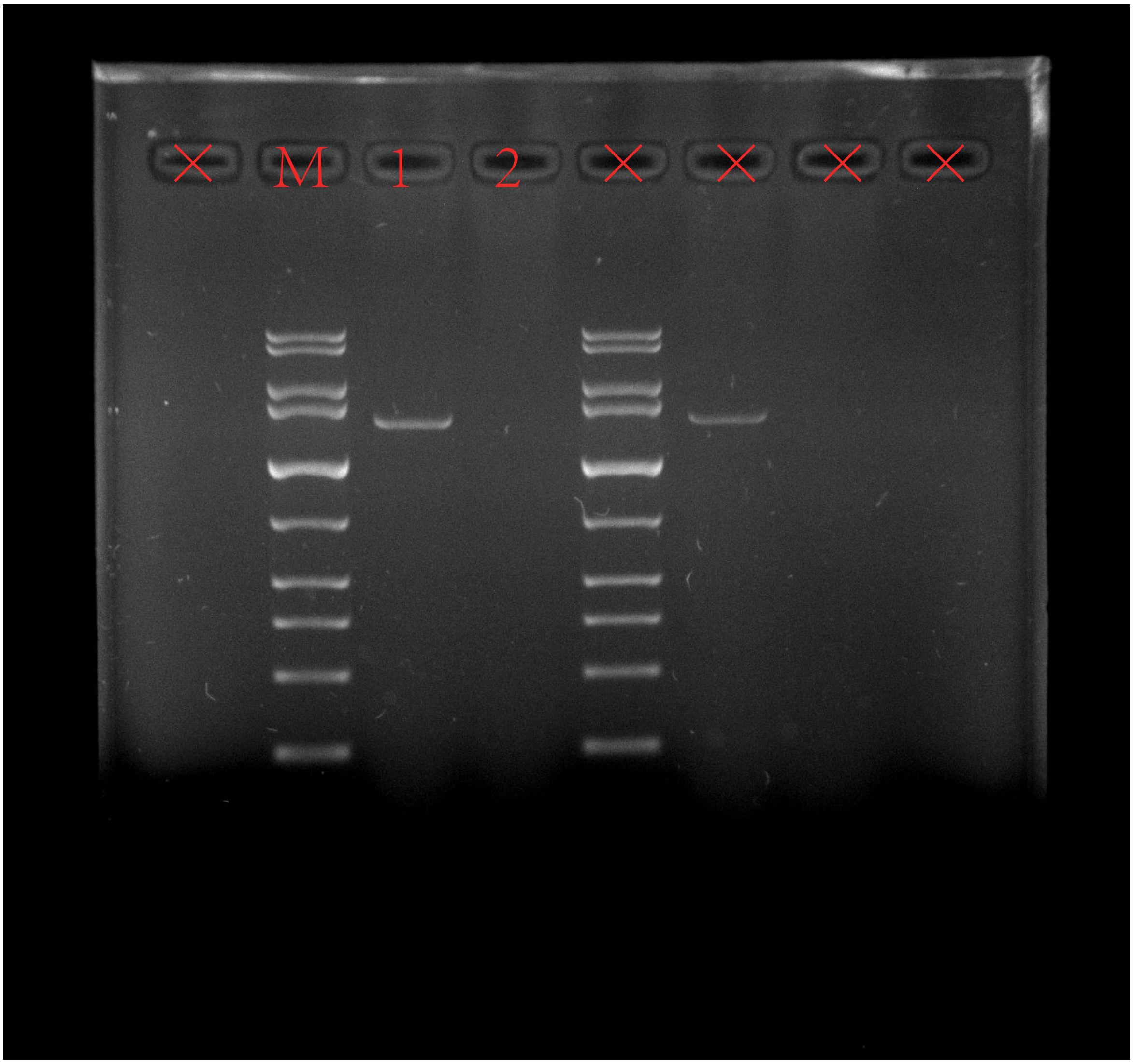

Figure 5B

Original Images for Gels

PCR products of the upstream homologous sequence of the *P5CR* gene + *hpt* gene sequence by electrophoresis

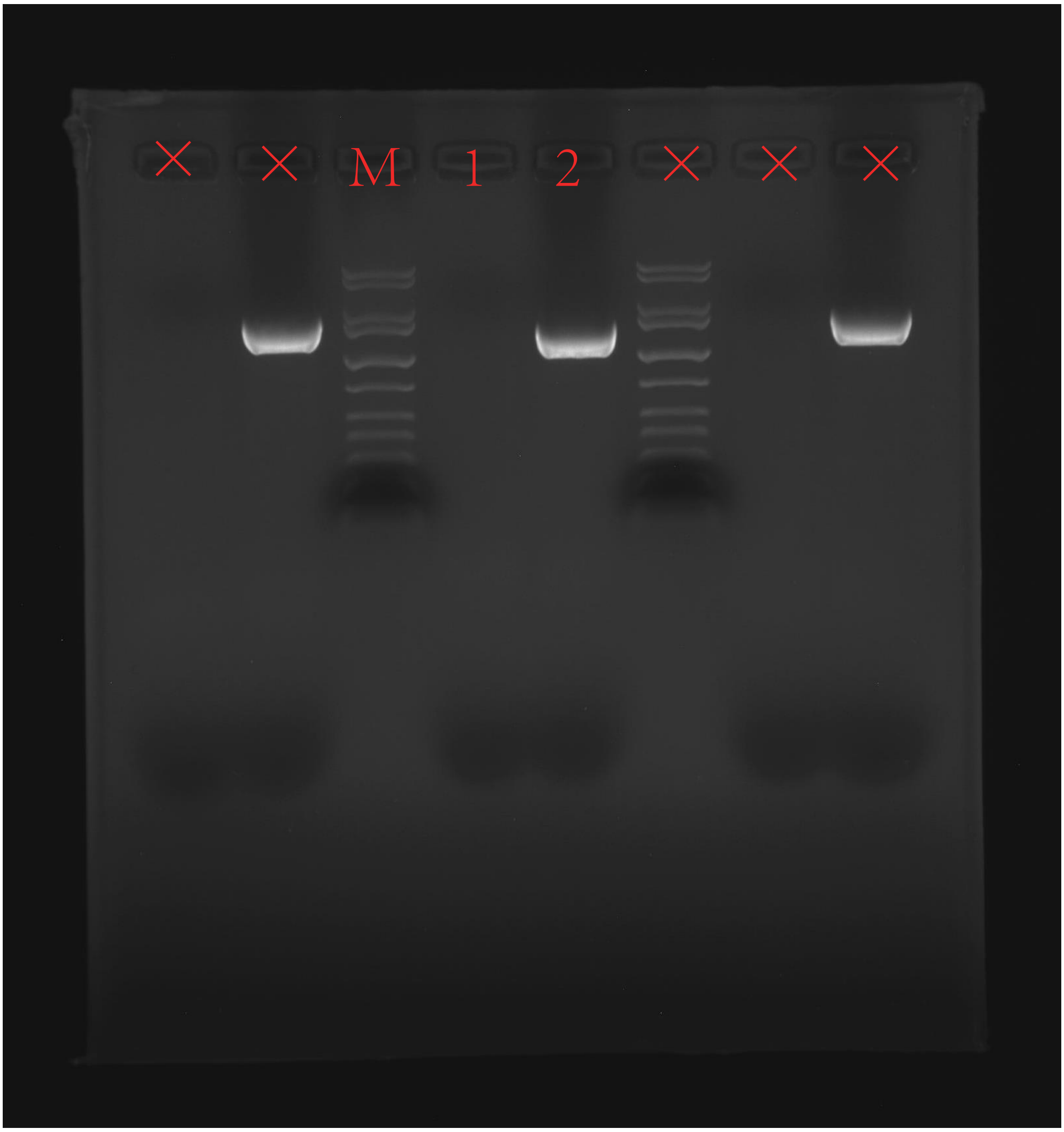

Figure 5C

Original Images for Gels

PCR products of the *P5CR* gene knockout cassette sequence by electrophoresis

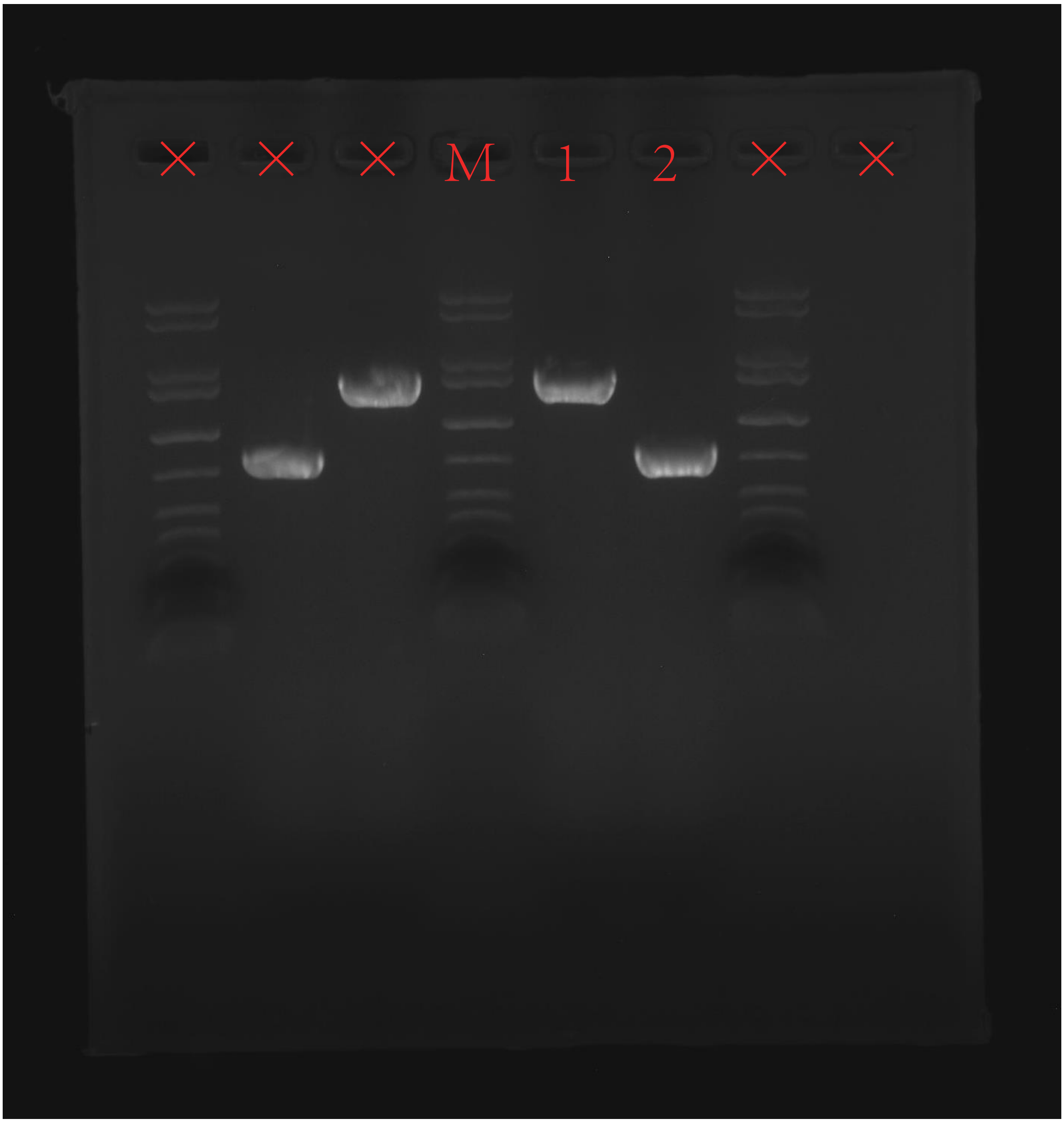

Figure 5D

Original Images for Gels

PCR products of the internal sequence of the *P5CR* gene by electrophoresis

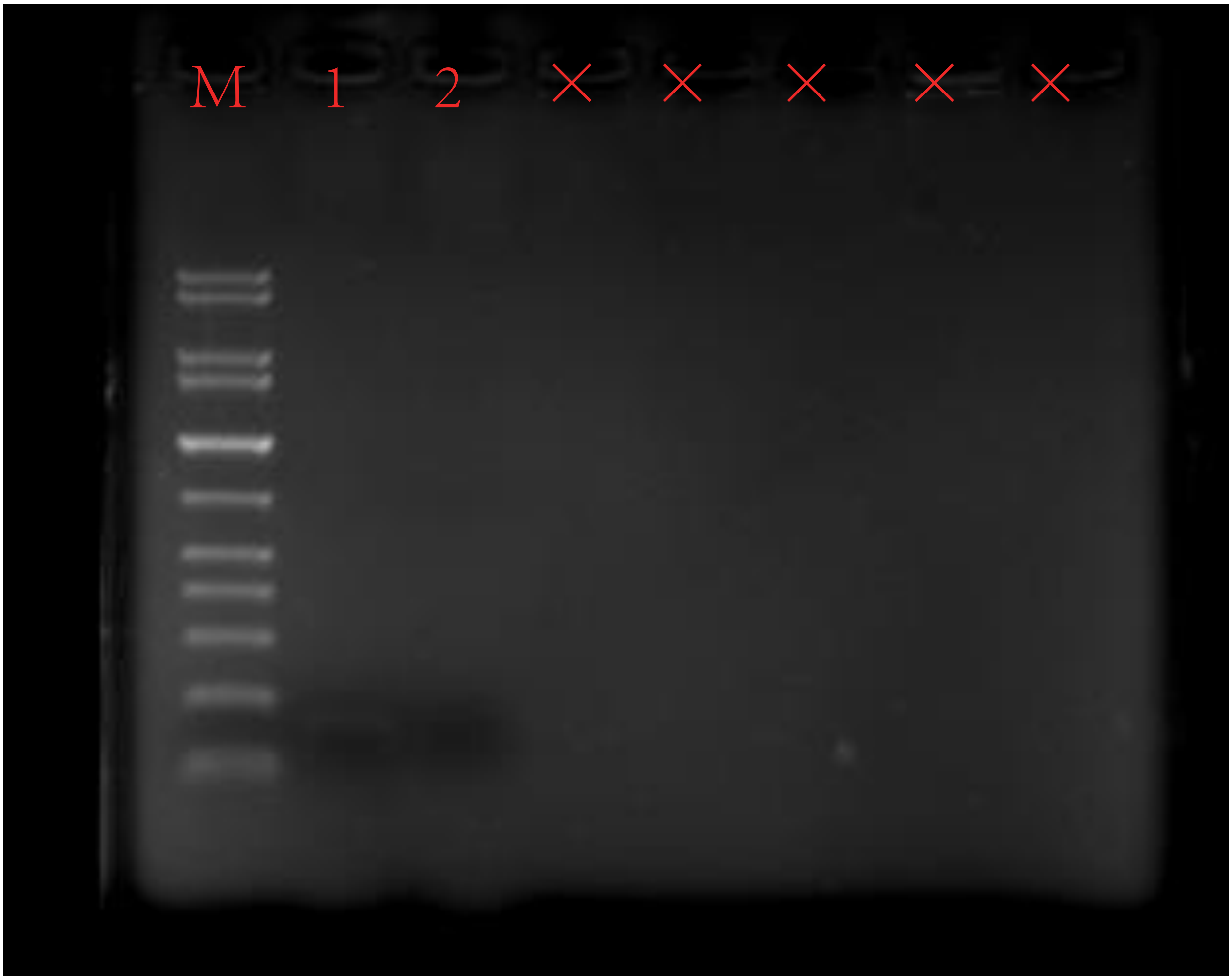

Supplement: Supplementary file 1 [file biomolecules-15-00460-s001.zip › biomolecules-3532710-supplementary.pdf]
